# Removal of Co(II), Cu(II) and Pb(II) ions by polymer based 2-hydroxyethyl methacrylate: thermodynamics and desorption studies

**DOI:** 10.1186/1735-2746-9-31

**Published:** 2012-12-22

**Authors:** Omid Moradi, Behrooz Mirza, Mehdi Norouzi, Ali Fakhri

**Affiliations:** 1 Department of Chemistry, Shahre-Qods Branch, Islamic Azad University, Shahre-Qods, Iran; 2 Department of Chemistry, South Tehran Branch, Islamic Azad University, Tehran, Iran; 3 Department of Pathobiology, School of Public Health, Tehran University of Medical Sciences, Tehran, Iran

**Keywords:** Removal, Polymeric surfaces, Langmuir isotherm, Heavy metal ions, Thermodynamics parameters

## Abstract

Removal thermodynamics and desorption studies of some heavy metal ions such as Co(II), Cu(II) and Pb(II) by polymeric surfaces such as poly 2-hydroxyethyl methacrylate (PHEMA) and copolymer 2-hydroxyethyl methacrylate with monomer methyl methacrylate P(MMA-HEMA) as adsorbent surfaces from aqueous single solution were investigated with respect to the changes in pH of solution, adsorbent composition, contact time and temperature in the individual aqueous solution. The linear correlation coefficients of Langmuir and Freundlich isotherms were obtained and the results revealed that the Langmuir isotherm fitted the experiment results better than Freundlich isotherm. Using the Langmuir model equation, the monolayer removal capacity of PHEMA surface was found to be 0.7388, 0.8396 and 3.0367 mg/g for Co(II), Cu(ΙΙ) and Pb(II) ions and removal capacity of P(MMA-HEMA) was found to be 28.8442, 31.1526 and 31.4465 mg/g for Co(II), Cu(ΙΙ) and Pb(II) ions, respectively. Changes in the standard Gibbs free energy (ΔG^0^), standard enthalpy (ΔH^0^) and standard entropy (ΔS^0^) showed that the removals of mentioned ions onto PHEMA and P(MMA-HEMA) are spontaneous and exothermic at 293–323 K. The maximum desorption efficiency was 75.26% for Pb(II) using 0.100 M HNO_3_, 70.10% for Cu(II) using 0.100 M HCl, 59.20% for 0.100 M HCl 63.67% Co(II).

## Introduction

Heavy metals such as lead, chromium, cobalt and copper are naturally occurring elements (Rafati *et al.*, [1,2]). Small amounts of these elements are common in our environment and they are actually necessary for our health. But large amounts of any of them may cause acute or chronic toxicity [3]. Heavy metals in human bodies tend to bioaccumulation, which may result in damaged or reduced mental and central nervous function, and damage to blood composition, lungs, kidneys and liver. As many heavy metal salts have high solubility in water, many different treatment techniques such as chemical precipitation, coagulation–precipitation, removal and ion exchange have been developed to remove heavy metals from contaminated water.

Several methods have been applied during many years for the elimination of these metal ions present in industrial wastewaters. The commonly traditional methods used for removal of heavy metal ions from aqueous solution include ion- exchange, solvent extraction, chemical precipitation [3], nano- filtration [4], reverse osmosis and removal [5].

Precipitation methods are particularly reliable but require high installation cost (large settling tanks for the precipitation) and usually a further treatment is also needed, in order to meet the law requirements. Removal, which is a more sophisticated technique, has the advantage of allowing the recovery of metallic ions, though is sometimes more expensive than the other techniques. Studies on the treatment of effluents containing heavy metals have revealed the removal to be a highly effective technique for removal of heavy metals from wastewater. Additionally, removal has been of the initial cost, simplicity of design and easiness of operation.

Copper ions are of particular interest because of its toxicity and widespread presence in the industrial applications, e.g. electrical, electro-plating, metal-finishing and paint industries. The toxicity of copper may cause itching and dermatitis, keratinization of the hands, and the soles of the feet (Al-Asheh and Banat, [6,7]). Therefore, the concentration of this ion must be reduced to the levels satisfying environmental regulations for various bodies of water. Lead ions are one of the major environmental pollutants. It is mainly discharged from exhaust gases of automobiles to the environment [8]. Moreover, it diffuses to the water and environment through effluents from lead smelters, battery manufacturers, paper and pulp industries and ammunition industries [8].

In recent years, various adsorbents have been used for removal of Co(II), Cu(II) and Pb(II) ions from aqueous solution. However, new adsorbents which are locally available, have high removal capacity and are economic still are needed [9,10]. Several authors have reported studies on various adsorbents. A number of adsorbent materials such as activated carbon derived from fertilizer waste, tea factory waste [11], goethite [12], amorphous iron oxide [13], kaolinite (Bhattacharyya and Gupta*.*, [14]), phenolated wood resin [15] modification of cellulose [16], zeolites [17], egg Shell [18], hydrogle [19] and modified jute [20] have been used in heavy metal removal from wastewaters. The obvious advantage of removal method is the lower costs involved. Hence, there is a need to search for more economical and effective adsorbents [21].

Hydrogels that are cross-linked hydrophilic polymers have been widely used in application field from agriculture to controlled delivery systems [22], removal of protein in medicine application and removal some ions from aqueous solution for environmental application and wastewater [23,24]. Among the potential adsorbents for removal of heavy metal ions such as Co(II), Cu(II) and Pb(II) ions, polymeric adsorbents with high surface area and pore structure have proved to be the promising candidates [25]. Tian showed that synthetic polycationic polymers were capable of removal more than 99.5% of the nitrate ions from aqueous solutions [26]. Salin has illustrated that for the effective removal heavy metals ions, Poly (EGDMA/HEMA) can be used [27]. Polymers which can selectively adsorb metal ions should consist of two monomer groups, each having a different role. One group forms a complex with the target (removal part) and the other allows the polymers to stretch and shrink reversibly in response to environmental change (the responsive part). Generally, 2-hydroxyethyl methacrylate is chosen as the responsive monomer. Polymers with interpenetration network structure were also studied to investigate removal of heavy metal ions (Abou, *et al*., [28]).

There are various possible interaction effects between different species in solution and the surface depends on the removal mechanism. Factors that affect the preferences for an adsorbate may be related to the characteristics of the binding sites (e.g. functional groups, structure, surface properties, etc.), the properties of the adsorbent (e.g. concentration, ionic size, ionic weight, ionic charge, molecular structure, ionic nature or standard redox potential, etc.) and solution specifications (e.g. pH, ionic strength, etc.) [29,30].

The objective of the present work is to investigate the removal potential of PHEMA and P(MMA-HEMA) surfaces for removal of Co(II), Cu(ΙΙ) and Pb(II) ions in the individual aqueous solution. Firstly (PHEMA and P(MMA-HEMA)) surfaces were synthesized; these surfaces have the potential for biodegrading by environment; then the effect of pH, HEMA/MMA ratio, contact time and temperature on the removal capacity of PHEMA and P(MMA-HEMA) surfaces were studied. The Langmuir and Freundlich isotherm models were used to describe equilibrium data. The removal mechanisms of Co(II), Cu(ΙΙ) and Pb(II)ions from aqueous solution onto PHEMA and P(MMA-HEMA) surfaces were also evaluated in terms of thermodynamic. Also, in order to evaluate the project from the economic point of view, desorption efficiency was also studies.

## Materials and methods

### Characterization of adsorbent

The materials used in the experiments were as follows: The MMA (molecular weight 100.12 g/mole, from Sigma company), and HEMA (molecular weight 130.14 g/mole, from Sigma company), which were used the monomers to synthesis the polymers in the presence of ammonium peroxo disulfate (APS), and sodium disulfite (SDS) as initiator, ethylene glycol dimethacrylate (EGDMA) as cross-link agent (all from Merck company). For synthesis of poly (HEMA), 99.5% percent weight HEMA monomer, 0.5% percent weight EGDMA as cross-link agent, APS and SDS as initiators were used. Also for synthesis of poly (MMA-HEMA), weight percentage of MMA and HEMA monomers was changed. Surfaces with (1%MMA-HEMA), (2%MMA-HEMA), (3%MMA-HEMA), (4%MMA-HEMA) and (5%MMA-HEMA), were prepared. The amount of EGDMA was fixed at 0.5%, than APS and SDS used as initiators for all surfaces. All surfaces were dried at 105°C for 24h, then washed with distilled water several times to remove dust and other water-soluble impurities. The prepared surfaces were similar to other referenced [31-34]. P(HEMA) and P(MMA-HEMA) were cut in 1 cm of diameter and 0.5 mm thick pieces. It is notable that, PHEMA has neutral surface charge, but P(MMA-HEMA) has negative charge, because MMA is a polar monomer [31].

### Removal procedure

Cu(NO_3_)_2_.3H_2_O (molecular mass: 241.60g/mol; CAS Number: 10031-43-3) , Co(NO_3_)_2_·6H2O (molecular mass: 182.943g/mol; CAS Number: 10026-22-9) and Pb(NO3)_2_ (molecular mass: 331.20g/mol; CAS Number: 10099-74-8) were used. Sodium dihydrogen phosphate, phosphoric acid, ammonium acetate, acetic acid, ammonium chloride and ammonia were used for buffer solution. All components were prepared from Merck Company with purity more than 99.99%.

Initial solutions with different concentration of Cu(II), Co(II) and Pb(II) ions were prepared by proper dilution from 1000mg/L standards. Removal process was carried out in 100 mL of ion containing solutions. For determination of removal of ions onto the surfaces, the difference between the initial and the equilibrium ions concentration by atomic absorbance spectrophotometry AAS (Perkin-Elmer AAnalyst 700) were measured (±0.01%). The concentrations of the adsorbed ions onto surfaces were determined through a calibration curve for the known ions concentration in the individual aqueous solution.

The surfaces (PHEMA and P(MMA-HEMA)) were treated by ion solution individually and contents in the sample solution were shaken for the desired contact time in an electrically thermostatic shaker at 150 rpm for all experiments. In this work, the contact time was varied from 10 to 160 min; the pH of the solution from 2 to 10, and the initial metal concentration were 10, 20, 30, 40 and 50 mg/L. Sodium phosphate buffer (0.1 mol/L) was prepared by adding an appropriate amount of phosphoric acid to sodium dihydrogen phosphate solution to make a solution with pH=2. Ammonium acetate buffers (0.l mol/L) were prepared by adding an appropriate amount of acetic acid to ammonium acetate solutions to result in solutions with pH=4-6. Ammonium chloride buffer solutions (0.l mol/L) were prepared by adding an appropriate amount of ammonia to ammonium chloride solutions to result in solutions of pH=7-9 [35].

Quality assurance of the analytical measurements was performed. Cu(II), Co(II) and Pb(II) standard solutions of 1000 mg/L ±0.1% were used for measurements. Calibration curves between 1 and 50mg/L were prepared, and the detection limit was found to be 1mg/L. Precision of the parallel measurements was ±1% SD. In all experiments double distilled deionized water (Milli-Q treated) and pH meter (M-12) from HORIBA Company for control pH solution (±0.01) were used. For determination of thermodynamics parameters, removal of Cu(II), Co(II) and Pb(II) ions by PHEMA and P(3%MMA-HEMA) surfaces at pH=6 versus different temperatures were studied. Then equilibrium constants obtained at 293, 303, 313 and 323K were used to determine the thermodynamics parameters. In order to study the desorption, different concentrations of mineral acids, as 0.01–0.100 M HNO_3_ for Pb(II) and 0.01– 0.100 M HCl for Co(II) and Cu(ΙΙ) ions were used as desorbing media for the desorption studies. The temperature was set at 298K. The data analysis was carried out using correlation analysis employing least-square method, and the average relative error (ARE) was calculated using the following equation:

(1) $$\frac{100}{n}\sum_{i=1}^{n}\left|\frac{q_{e,\exp}-q_{e,\mathrm{calc}}}{q_{e,\exp}}\right|$$

where n is the number of data points, *q*_*e*_ is the amount adsorbed at equilibrium and the subscripts ‘exp’ and ‘calc’ show the experimental and calculated values, respectively. Each experiment was conducted in triplicate under identical conditions to confirm the results and was found reproducible.

### Equilibrium

The removal data can then be correlated with Langmuir and Freundlich isotherm model equations. If ion removal follows linear Langmuir equation, the removal can be expressed as: [36]

(2) $$\frac{1}{q_{e}}=\frac{1}{q_{m}}+\frac{1}{q_{m}K_{L}}\frac{1}{C_{e}}$$

where *ss*_*L*_ is the removal equilibrium constant (L/mg), *q*_*m*_ is the maximum removal capacity (mg/g), *C*_*e*_ the equilibrium metal ion concentration (mg/L), and *q*_*e*_ is the amount of adsorbed ion at equilibrium (mg/g). The Freundlich model in linear form is [37]:

(3) $$\ln q_{e}=\ln K_{f}+\frac{1}{n}\ln C_{e}$$

where *K*_*f*_ and *n* are the Freundlich constants.

### Thermodynamic parameters

The thermodynamic parameters of the removal, i.e. the standard enthalpy change, ΔH^0^, the Gibbs free energy change, ΔG^0^ and the standard entropy change, ΔS^0^ were calculated using equations (Adamczyk and Warszyhski , [38,39]):

(4) $$\Delta G^{0}=-\mathrm{RT}\ln K_{o}$$

(5) $$K_{0}=\frac{q_{e}}{C_{e}}$$

(6) $$\ln K_{0}=-\frac{\Delta H^{0}}{R}\frac{1}{T}+\frac{\Delta S^{0}}{R}$$

where T is temperature in Kelvin and R is the universal gas constant (8.314 J/mol K). The thermodynamic equilibrium constant was determined by Equation 6 at different temperatures and then was concluding the values of ΔH^0^ and ΔS^0^[40].

### Desorption studies

Desorption studies were carried out to understand the regenerative capability of PHEMA and P(3%MMA-HEMA) surfaces. Different concentrations of mineral acids, as 0.01–0.100 M HNO_3_ for Pb(II) and 0.01–0.100 M HCl for Co(II) and Cu(ΙΙ) ions were used as desorbing media for the desorption studies. The temperature was set at 298 K. Desorption studies were performed maintaining the process conditions similar to those of removal studies [41].

## Results

### Effect of contact time

**Figure 1:**
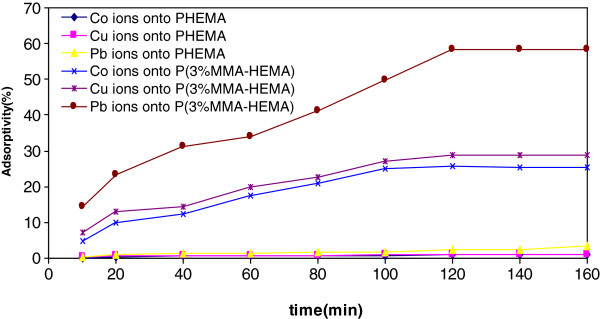
Effect of contact time on the removal of Cu(II), Co(II) and Pb(II) onto PHEMA and P(3%MMA-HEMA) surfaces (metal ions concentration=10 mg/L; pH=6; T=303±1K).

Effect of contact time on metal ions removal by PHEMA and P(1%MMA-HEMA) were studied by variation of the contact time (10 to 160 min) for constant initial concentrations(10 mg/L). Figure 1 shows the removal percentages of Co(II), Cu(ΙΙ) and Pb(II) ions as a function of contact time. 

### Effect of adsorbent composition

**Figure 2:**
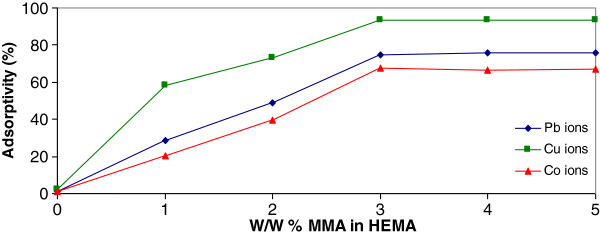
Effect of adsorbent composition on the removal of Cu(II), Co(II) and Pb(II) ions (metal ions concentration=10 mg/L; pH=6; T=303±1°K).

The effect of adsorbent composition on the removal percentage of Co(II), Cu(ΙΙ) and Pb(II) ions is shown in Figure 2. When MMA weight percentage was increased from 1 to 3, the removal percentage was raised from 0.95 to 20.35% for Co(II), 1.07 to 28.8% for Cu(II) ions and from 2.31 to 58.3% for Pb(II) ions. The removal percentage of metal ions at 3% W/W of MMA almost is maximized (for Co(II) 67.31% removal percentage, Cu(II) 74.5% removal percentage and Pb(II) 93.5% removal percentage ions) and with further increasing of MMA, removal percentage remains constant. 

### Effect of pH solution on removal

**Figure 3:**
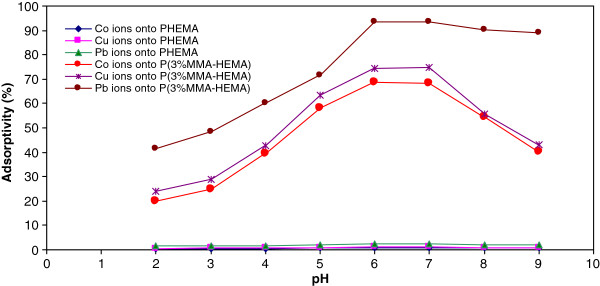
Effect of pH solution on Cu(II), Co(II) and Pb(II) ions removal onto PHEMA and P(3%MMA-HEMA) surfaces, (metal ions concentration=10 mg/L; T=303±1K).

Figure 3 shows the effect of pH on the removal of Co(II), Cu(ΙΙ) and Pb(II) ions onto PHEMA and P(3%MMA-HEMA) surfaces. The removal percentage was found to increase from 0.33 to 20.1% for Co(II) onto PHEMA and P(3%MMA-HEMA) surfaces, 0.52 to 23.9% for Cu(II) onto PHEMA and P(3%MMA-HEMA) surfaces and 1.45 to 41.5% for Pb(II) onto PHEMA and P(3%MMA-HEMA) surfaces, when pH was increased from 2 to 9 respectively. (Bruno and Svorons, [35]). 

### Effect of temperature on the removal

**Figure 4:**
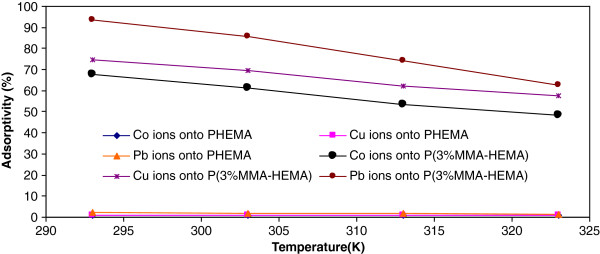
Effect of temperature on Cu(II), Co(II) and Pb(II) ions removal onto PHEMA and P(3%MMA-HEMA) surfaces, (metal ions concentration=10 mg/L; pH=6; T=303±1K).

Figure 4 shows the representative plots of isotherm removal percentages of Co(II), Cu(ΙΙ) and Pb(II) ions onto PHEMA and P(3%MMA-HEMA) surfaces versus different temperature ranging from 293 to 323K. In this section, concentration of ions was 10 mg/L, pH=6 and contact time was 120 min. It was found that the removal of ions with an increasing in temperature onto surfaces was decreased. When the temperature was increased from 293 to 323K, the removal percentage decreased from 0.94 to 0.69% for Co(II), 1.06 to 0.78% for Cu(II) ions and 2.31 to 1.45% for Pb(II) ions onto PHEMA surface, 67.80 to 48.31% for Co(II), 74.5 to 57.64% for Cu(II) ions and 93.5 to 62.78% for Pb(II) ions onto P(3%MMA-HEMA) surface at the equilibrium time.

### Effect of concentration on the removal

**Figure 5:**
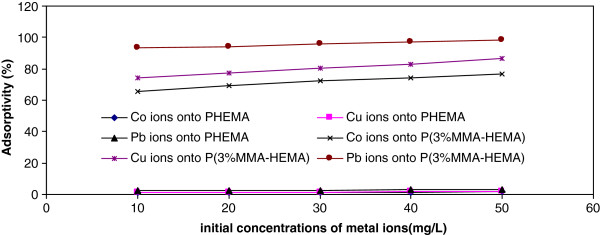
Effect of initial Cu(II), Co(II) and Pb(II) ions concentration on the removal onto PHEMA and P(3%MMA-HEMA) surfaces, (pH=6; T=303±1K).

Figure 5 shows the effect of initial concentrations on removal percentages of Co(II), Cu(ΙΙ) and Pb(II) ions onto PHEMA and P(3%MMA-HEMA) surfaces. The removal of Co(II), Cu(ΙΙ) and Pb(II)ions were carried out at different initial concentrations ranging from 10, 20, 30, 40 and 50 mg/L at pH 6, at 293 K with 120 min of contact time. The initial of ions (Co(II), Cu(ΙΙ) and Pb(II)) concentrations increased from 10 to 50 mg/L, the removal is increased (0.94 to 1.83% for Co(II), 1.06 to 2.05% for Cu(II) and 2.31 to 3.19% for Pb(II) onto PHMEA surface respectively; 65.56 to 76.8% for Co(II), 74.5 to 86.33% for Cu(II) and 93.5 to 98.1% for Pb(II) onto P(3%MMA-HEMA) surface respectively). 

### Equilibrium and thermodynamic parameters

Also, comparison between the Langmuir and Freundlich isotherm is presented in Table 1 and 2. As can be seen in this Table 1, the amount of for liner correction coefficient (R^2^) for Langmuir isotherm is less than Freundlich isotherm. So we can conclude that the Langmuir isotherm of the Freundlich isotherms is more suitable.

**Table 1:** Comparison between Langmuir and Freundlich isotherms

**Surfaces**	**Langmuir isotherm**		**Freundlich isotherm**	
	**(ARE)**	**R**^**2**^	**(ARE)**	**R**^**2**^
Co ions onto PHEMA	3.88	0.9995	7.35	0.9903
Cu ions onto PHEMA	3.34	0.9997	7.12	0.9915
Pb ions onto PHEMA	2.58	0.9997	7.00	0.9977
Co ions onto P(3%MMA-HEMA)	2.76	0.9978	7.02	0.9852
Cu ions onto P(3%MMA-HEMA)	2.01	0.9969	6.88	0.9744
Pb ions onto P(3%MMA-HEMA)	1.89	0.9958	6.44	0.9997

**Table 2:** Parameters of (a) Langmuir and (b) Freundlich removal isotherms for Co(II), Cu(II) and Pb(II) ions onto surfaces

**Surfaces**	**q**_**m**_**(mg/g)**	**K**_**L**_**(L/mg)**
Co ions onto PHEMA	0.7388	0.0099
Cu ions onto PHEMA	0.8396	0.0113
Pb ions onto PHEMA	3.0367	0.0072
Co ions onto P(3%MMA-HEMA)	28.8442	0.0660
Cu ions onto P(3%MMA-HEMA)	31.1526	0.0751
Pb ions onto P(3%MMA-HEMA)	31.4465	0.3412
**Surfaces**	**K**_**F**_	**1/n**
Co ions onto PHEMA	0.0034	1.13
Cu ions onto PHEMA	0.0039	1.40
Pb ions onto PHEMA	0.0139	1.21
Co ions onto P(3%MMA-HEMA)	0.1863	1.28
Cu ions onto P(3%MMA-HEMA)	1.5527	1.59
Pb ions onto P(3%MMA-HEMA)	2.6912	1.83

**Figure 6:**
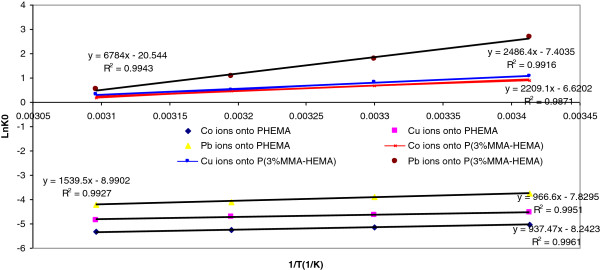
**lnK**_**0 **_**vs. 1/T plot.**

The results of Thermodynamic parameters are indicated in Figure 6 and summarized in Table 3. The equilibrium constants obtained from Langmuir isotherm at 293, 303, 313 and 323K were used to determine the Gibbs free energy changes. Table 3 shows the thermodynamic parameters values for the removal of Co(II), Cu(ΙΙ) and Pb(II) ions onto the surfaces 

**Table 3:** Thermodynamic parameters of Co(II), Cu(II) and Pb(II) ions removal onto surfaces

**Surfaces**	**ΔH**^**0**^**kJ/mol**	**ΔS**^**0**^**J/mol K**	***ΔG***_**293**_^**0**^**kJ/mol**	***ΔG***_**303**_^**0**^**kJ/mol**	***ΔG***_**313**_^**0**^**kJ/mol**	***ΔG***_**323**_^**0**^**kJ/mol**
Co ions onto PHEMA	−7.794	−68.52	−27.87	−28.55	−29.24	−29.92
Cu ions onto PHEMA	−8.036	−65.09	−27.10	−27.75	−28.40	−29.06
Pb ions onto PHEMA	−12.799	−74.74	−34.73	−35.45	−36.19	−36.94
Co ions onto P(3%MMA-HEMA)	−18.366	−55.04	−34.49	−35.04	−35.59	−36.14
Cu ions onto P(3%MMA-HEMA)	−20.672	−61.55	−38.70	−39.32	−39.94	−40.55
Pb ions onto P(3%MMA-HEMA)	−56.400	−17.08	−61.40	−61.58	−61.75	−61.92

### Desorption studies

Desorption studies were performed maintaining the process conditions similar to those of removal studies. The results of desorption studies are depicted in Table 4.

**Table 4:** Desorption of Co(II), Cu(IΙ) and Pb(II) ions onto PHEMA and P(3%MMA-HEMA) surfaces

**Concentration of acid (M)**	**Desorption of Pb(ІІ)%**	**Desorption of Cu(ΙΙ)%**	**Desorption of Co(ΙΙ)%**
0.010	19.28	16.17	14.02
0.025	41.15	31.22	28.47
0.050	50.48	42.47	35.34
0.075	67.32	59.87	50.68
0.100	75.26	70.10	63.67

## Discussion

### Effect of contact time

In order to optimize the contact time for removal, PHEMA and P(1%MMA-HEMA) surfaces were prepared. Then the surfaces were treated by Co(II), Cu(ΙΙ) and Pb(II) ions solutions with 10 mg/L of concentration, pH=6 and T=303±1K. It can be seen that the amounts of Co(II), Cu(ΙΙ) and Pb(II) ions adsorbed onto P(1%MMA-HEMA) are more than the amounts were adsorbed by PHEMA surface, since metal ions has positive charge and P(1%MMA-HEMA) has negative surface charge. It seems that attractive interaction has main role in removal process, since PHEMA has neutral surface [31]. Also with increasing contact time to 120 min, the percentage of removal was increased. After 120 min, the amount of adsorbed ions remained unchanged. Therefore, this duration was selected as the optimum contact time for all further experiments. The same equilibrium times have been reported in several earlier works which related with the removal of Co(II), Cu(ΙΙ) and Pb(II) ions on various adsorbents [42,43].

### Effect of adsorbent composition

The increase in the removal percentage with change in adsorbent composition is due to increasing in active site on the adsorbent and which makes penetration of the metal ions to the removal site much easier. Also attractive interaction increases between ions and adsorbent surface. When ions in solution contact another phase (solid, liquid, or a gas) which is immiscible, the ions tend to accumulate at the interface between two phases. This tendency has a great effect on various natural and technological processes. Removal of ions takes place almost instantaneously when a solid surface comes into contact with most aqueous solutions. Adsorbent composition is an important parameter in removal process. The main reason for this trend may be that the surfaces are fully coverage and saturated [44]. Therefore, the amount of P(3%MMA-HEMA) was selected for further removal experiments.

### Effect of pH solution on removal

The pH of solution is the most important variable affecting metal ions removal. This is partly because hydrogen ions themselves are strongly competing with metal ions. At low pH values, the low removal observation was explained due to electrostatic attraction occurred between P(3%MMA-HEMA) surface and metal ions. The P(3%MMA-HEMA) surface has negative charge and ions has positive charge. With increase in pH of solution, the amount of removal is increased until pH= 6–7 which shows maximum amount of removal for both surfaces. In all solution, competitive removal has been hydronium ions (H_3_O^+^) and other ions (Co(II), Cu(ΙΙ) and Pb(II)). At low values pH, hydronium ions more than other ions adsorbed. Because hydronium ions, has high concentration and more tendency for the removal [45]. But with increasing pH, hydronium ions concentration is reduced and to the result in more adsorbed other ions in solution [45,46]. A considerable increase in the removal was occurred at pH=6-7 and the maximum Co(II), Cu(ΙΙ) and Pb(II) ions removals were observed at the almost pH=6-7. At higher pH values, metal precipitation appeared and adsorbent was deteriorated with accumulation of metal ions onto surfaces [46]. Therefore, pH=6 was selected as the optimum pH for further studies. A similar theory was proposed by several earlier workers for metal ions removal on different adsorbents [45].

The results for PHEMA surface is similar to P(3%MMA-HEMA) surface, but PHEMA surface has neutral charge and other variable interaction such as hydrophobicity surface has effect on the removal (Martinez *et al*., [47]).

### Effect of temperature on the removal

Temperature has a pronounced effect on the removal capacity of adsorbents. A decrease in the removal for ions with the rise in temperature may be explained by being more active adsorbent sites at low temperature (Berber- Mendoza *et al*., [48]). Also an increase in temperature result in an increased mobility of the ions and a decrease in the retarding forces acting on the removal ions Berber -Mendoza *et al*., [48]). This result may also confirm the exothermic nature of Co(II), Cu(ΙΙ) and Pb(II) ions removal onto PHEMA and P(3%MMA-HEMA) surfaces. So 293K was selected as the solution temperature. Similar trends have been observed for the removal of other heavy metal ions.

### Effect of concentration on the removal

The results may be explained by the fact that, at selected pH, the surface of adsorbent would also surrounded by hydronium ions which enhance ions interactions with binding site of the adsorbent by greater attractive force. As the initial concentrations increased, the removal percentage is increased.

### Equilibrium and Thermodynamic parameters

Furthermore, ΔS^0^ and ΔG^0^ changes should be considered for determining occurs spontaneous of process. The Gibbs free energy change indicates the degree of spontaneously of removal process and higher negative value reflects a more energetically favorable removal [40]. The standard enthalpy changes were determined −7.794, -8.036, -12.799 kJmol^-1^ for Co(II), Cu(ΙΙ) and Pb(II) ions onto PHEMA surface respectively,-18.36, -20.672, -56.40 kJmol^-1^ for Co(II), Cu(ΙΙ) and Pb(II) ions onto P(3%MMA-HEMA) surface respectively from Figure 6. Also the standard entropy changes were determined −68.52, -65.09, -74.74 J/molK for Co(II), Cu(ΙΙ) and Pb(II) ions onto PHEMA surface respectively, -55.04, -61.55, -17.08 Jmol^-1^K^-1^ for Co(II), Cu(ΙΙ) and Pb(II) ions onto P(3%MMA-HEMA) surface respectively from Figure 6. A negative the standard enthalpy change obtained in this study indicates that the removal of both ions by PHEMA and P(3%MMA-HEMA) surfaces is exothermic, which is evidenced by the decrease in the removal of both ions with temperature increase. A negative change in ΔG^0^ reveals that the removal reaction is spontaneous (Do *et al*., [49]). The standard entropy change was found to be negative values for those processes. This mains a decrease in the randomness at solid-solution interface during the removal of Co(II), Cu(ΙΙ) and Pb(II) ions onto PHEMA and P(3%MMA-HEMA) surfaces. The results are similar to previous literatures [50,51]. With attention this point PHEMA and P(3%MMA-HEMA) surfaces is used as products and adsorbent for purification of water, also their products (PHEMA and P(3%MMA-HEMA) surfaces) is not toxic for human and possible to biodegrading.

### Desorption studies

It is evident from Table 4 that the maximum desorption efficiency was 75.26% for Pb(II) using 0.100 M HNO_3_, 70.10% for Cu(II) using 0.100 M HCl and 63.67% for Co(II) using 0.100 M HCl. Hydronium ions may replace Co(II), Cu(ΙΙ) and Pb(II) ions on the metal loaded adsorbent, thus functioning as a cation exchanger. The metal ions loaded on PHEMA and P(3%MMA-HEMA) surfaces create disposal problem as they are hazardous in nature. This problem may be overcome to some extent by using elution methods. Elution of heavy metals allows the recovery of metal ions in concentrated solutions and regenerated adsorbents. Concentrated metal solutions may be suitable for metal recovery. Regenerated adsorbents may be recycled for reuse and ultimately the adsorbents must be incinerated [41]. The evaluate this experimental from the economic is possible reuse surfaces adsorbent and hygienic of view is nontoxic PHEMA and P(3%MMA-HEMA) surfaces for human and environments.

## Competing interests

The authors declare that they have no competing interests.

## Authors’ contributions

OM and AF carried out the isotherms and thermodynamic studies and participated in the drafted the manuscript. BM and MN carried out the effect of different parameters studies. All authors read and approved the final manuscript.
